# John Mackinder

**Published:** 1822-09

**Authors:** 


On Sunday last, (August 25th,) after only three days' illness, at his house in Middlesex-place, New-road, St. Mary-le-bone, John Mackinder, Esq. surgeon, for many years an able practitioner in that neighbourhood.

